# Emerging, novel gene-modulating therapies for transthyretin amyloid cardiomyopathy

**DOI:** 10.1007/s10741-025-10502-5

**Published:** 2025-03-08

**Authors:** Song Peng Ang, Jia Ee Chia, Debabrata Mukherjee

**Affiliations:** 1 Department of Medicine, Rutgers Health/Community Medical Center, Toms River, NJ USA; 2 Department of Medicine, Texas Tech University Health Science Center, El Paso, TX USA; 3 Department of Cardiovascular Medicine, Texas Tech University Health Science Center, El Paso, TX USA

**Keywords:** Transthyretin amyloid cardiomyopathy, Gene-modulating therapies, CRISPR-Cas9, RNA interference, Anti-sense oligonucleotides

## Abstract

Transthyretin amyloid cardiomyopathy (ATTR-CM) is a progressive, life-threatening disease caused by the pathological deposition of misfolded transthyretin (TTR) protein in the myocardium, leading to restrictive cardiomyopathy and heart failure. While TTR stabilizers such as tafamidis and acoramidis are the only FDA-approved treatments, novel gene-modulating therapies are emerging as transformative approaches. Small interfering RNA (siRNA) and antisense oligonucleotide (ASO) therapies effectively reduce TTR production and have demonstrated promising clinical outcomes, though their use in cardiac amyloidosis remains investigational. CRISPR-Cas9 therapies represent a paradigm shift, offering a potential one-time treatment by permanently silencing the TTR gene. Recent clinical trials have shown significant TTR reduction and stabilization of disease biomarkers, although long-term safety and efficacy require further evaluation. Despite the lack of direct comparisons among these modalities, their emergence highlights a promising future for ATTR-CM management. This review discusses the pathogenesis of ATTR-CM, mechanisms of novel gene-modulating therapies, clinical evidence, challenges, and the future outlook for advancing treatment options.

## Introduction

Transthyretin amyloid cardiomyopathy (ATTR-CM) is a progressive disease characterized by the extracellular deposition of transthyretin (TTR) protein in the myocardium [1, 2]. This condition leads to restrictive cardiomyopathy, impaired diastolic function, and ultimately, heart failure [3]. ATTR-CM primarily exists in two forms: wild-type ATTR-CM (wtATTR), often associated with aging, and variant ATTR-CM (ATTRv), caused by mutations in the TTR gene [2]. Despite the availability of therapies like TTR stabilizers, the disease remains challenging to treat due to its progressive nature and limited options to reduce existing amyloid deposits [4].

The emergence of gene-modulating therapies represents a paradigm shift in the management of ATTR-CM [5]. Techniques like small interfering RNA (siRNA) therapy and CRISPR-Cas9 gene editing directly target the underlying cause of the disease by suppressing or modifying the TTR gene, effectively halting TTR production at its source [6–8]. siRNA therapies have demonstrated impressive reductions in circulating TTR levels, accompanied by reduction in mortality and cardiovascular events as well as clinical improvements in biomarkers (NT-proBNP and troponin) and cardiac function including left ventricular wall thickness and improved diastolic function [6, 9, 10]. Meanwhile, CRISPR-Cas9-based approaches are pioneering interventions that permanently disrupt TTR production, offering the potential for durable disease stabilization.

This review explores the molecular basis of ATTR-CM, the mechanisms of different gene modulating anti-ATTR therapies, their challenges, and the clinical evidence supporting their use.

## Brief pathogenesis of ATTR-CM

TTR is a tetrameric protein primarily synthesized in the liver, which is physiologically responsible for transporting vitamin A or retinol and thyroxine [11]. ATTR-CM is a form of amyloidosis caused by the deposition of misfolding and aggregation of TTR protein in the cardiac tissue. In ATTR-CM, instability of the TTR tetramer results in its dissociation into monomers, which misfold and aggregate into insoluble amyloid fibrils [3]. These fibrils accumulate extracellularly within the myocardium and cause disruption of the cardiac architecture and function.

There are two forms of ATTR-CM-wtATTR and ATTRv. wtATTR-CM occurs without TTR gene mutations and is more prevalent in elderly individuals, predominantly affecting males. In contrast, ATTRv is caused by pathogenic mutations in gene encoding the TTR protein, leading to earlier disease onset and variable phenotypic expression. The underlying TTR mutations drive a phenotypic spectrum, ranging from predominantly neuropathic to cardiac manifestations, with different variants exhibiting mixed involvement [12]. For instance, V30M (valine to methionine substitution at position 30) mutation classically presents with early-onset polyneuropathy with autonomic dysfunction, though late-onset cases often concomitantly demonstrate cardiac involvement [13, 14]. In contrast, V122I (valine to isoleucine substitution at position 122) often manifests as restrictive cardiomyopathy with minimal neuropathy, showing particular prevalence in African American populations [15]. This genotypic-phenotypic variability stems from the differences in amyloid fibril structure, tissue tropism, and gene effects modifier [16, 17].

## Antisense oligonucleotides (ASO)

### Mechanism of entry

ASOs enter hepatocytes primarily through receptor-mediated endocytosis [18, 19]. Eplontersen is conjugated by N-acetylgalactosamine (GAINAc), which binds to asialoglycoprotein receptor (ASGPR) on hepatocytes and triggers clathrin-mediated endocytosis [18]. ASGPR’s high expression and rapid recycling enable efficient hepatocyte-specific delivery [19].

### Mechanism of action

**Fig. 1:**
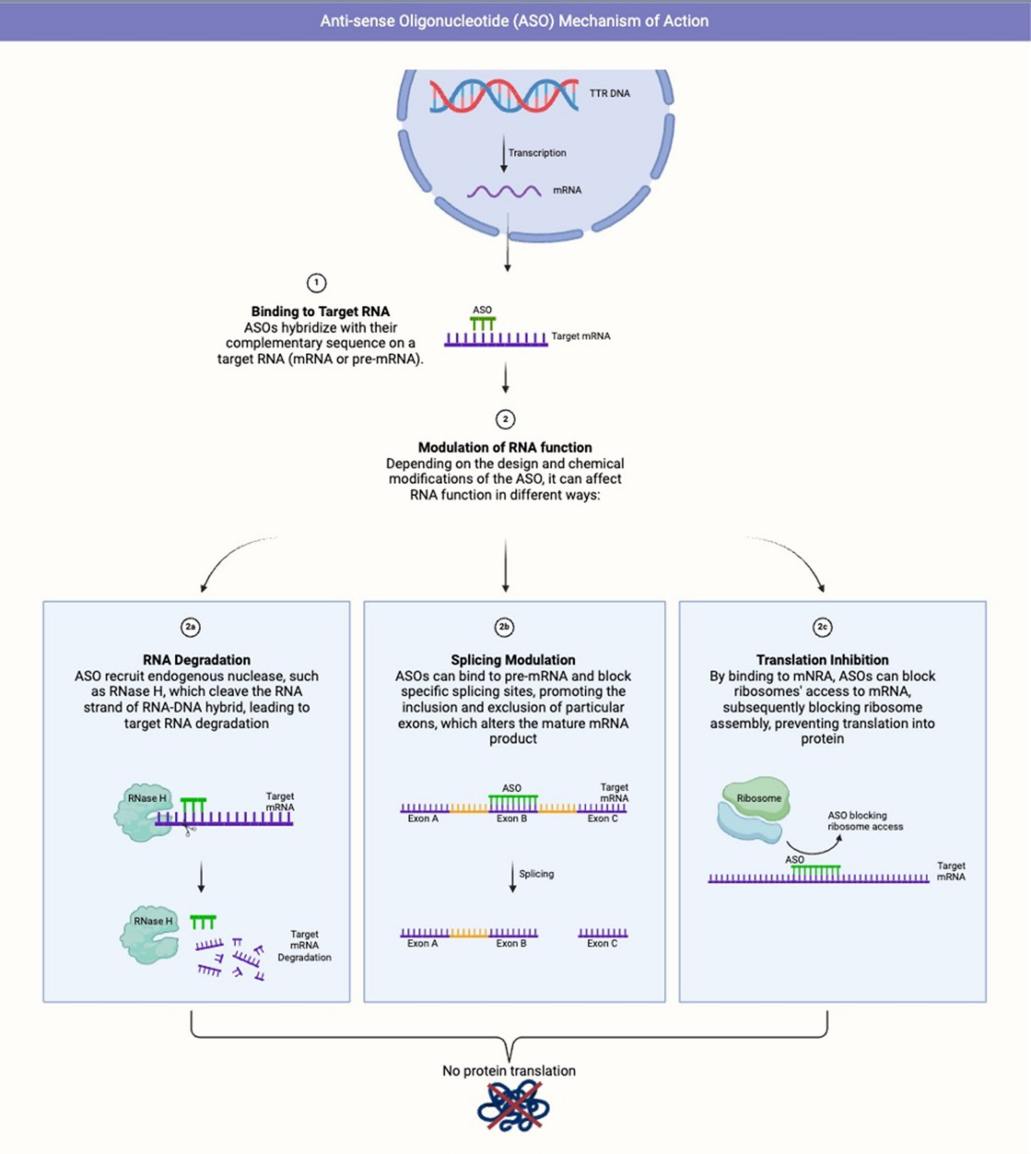
Mechanism of action of antisense oligonucleotides (ASOs)

ASOs target TTR mRNA within hepatocytes (Fig. 1) [19]. ASOs bind specifically to TTR mRNA, triggering its degradation via RNase H-mediated cleavage, thereby inhibiting TTR protein synthesis. By reducing expression and synthesis of both mutant and wild-type TTR, ASOs prevent the formation and deposition of amyloid fibrils in tissues.

### Inotersen

Inotersen was the first ASO therapy evaluated in the NEURO-TTR trial (ClinicalTrials.gov Identifier: NCT01737398), a pivotal phase 3 study involving 172 patients with variant transthyretin amyloidosis and polyneuropathy (ATTRv-PN) [20]. This 15-month, randomized, double-blind, placebo-controlled trial showed that weekly inotersen significantly improved neurological function and quality of life. Patients receiving inotersen showed a significant reduction in disease progression on the mNIS + 7 scale and a significant improvement in the Norfolk QOL-DN score with benefits consistent across disease stages, mutation types, and cardiomyopathy status [20]. Notably, however, patients with preexisting cardiomyopathy (*n* = 75, 67%) showed no statistically significant changes in cardiac parameters, including global longitudinal strain, septal thickness, posterior wall thickness, left ventricular ejection fraction, and lateral E/e′ ratio, from baseline through week 65 [20]. Safety concerns included thrombocytopenia (3% incidence) and glomerulonephritis (3%), leading to enhanced monitoring protocols, though five deaths occurred in the treatment group (one linked to severe thrombocytopenia). As a result of this study, in 2018, inotersen became the first ASO approved by the European Commission as well as the US Food and Drug Administration (FDA) for treatment of patients with ATTRv-PN [21].

Following that, a phase 2 clinical trial (ClinicalTrials.gov Identifier: NCT03702829) was carried out to study the tolerability and efficacy of inotersen in ATTR-CM patients [22]. However, inotersen was later withdrawn in September 2024 from the market voluntarily by its manufacturer, citing low utilization rate and emphasizing that this discontinuation was not due to safety reasons [23].

### Eplontersen

Eplontersen was primarily studied in the NEURO-TTRansform trial (ClinicalTrials.gov Identifier: NCT04136184; EU Clinical Trials Register: EudraCT 2019–001698-10, a phase 3, open label, multicenter trial involving patients with ATTRv-PN [24]. Masri and colleagues conducted a secondary analysis, focusing on the effect of eplontersen on the cardiac structure and function of these patients [25]. In summary, they compared the cohort from the NEURO-TTRansform trial as the intervention group and a placebo cohort from the NEURO-TTR trial (ClinicalTrials.gov number, NCT01737398) [20, 24]. Details of the study design have been described previously [20]. Here, we described the results of the subgroup analysis focused on patients with cardiomyopathy. In this subgroup, 49 patients received eplontersen while 30 patients received placebo. Compared with placebo, treatment with eplontersen was associated with an improvement in left ventricular ejection fraction (mean difference, 4.3%; 95% CI, 1.40–21.01; *P* = 0.049) and stroke volume (mean difference, 10.64 mL; 95% CI, 3.99–17.29; *P* = 0.002), measured from baseline to week 65.

### Challenges of ASO therapy

The initial challenge of ASO uptake into hepatocytes was related to their non-specific biodistribution. Unconjugated ASOs typically exhibit low hepatocyte specificity, with preferential accumulation in non-parenchymal liver cells (such as Kupffer cells) due to interactions with scavenger receptors and protein-binding properties of the phosphorothioate backbone [26]. This reduces efficacy for hepatocyte-specific RNA targets. The integrated safety analysis from 7 phase 2 studies for GalNAc-conjugated ASOs demonstrates that ligand-mediated targeting overcomes these challenges while maintaining safety [27]. As mentioned earlier, GalNAc conjugation redirects ASOs to hepatocytes via ASGPR, achieving at least 80% hepatic uptake compared to < 20% for unconjugated ASO [28]. This also reduced off-target accumulation in other organs such as the kidneys [29].

## Small interfering RNA (siRNA) therapy

### Mechanism of entry

siRNA delivery uses lipid nanoparticles (LNPs) or GalNAc conjugation for hepatocyte targeting [18]. In the case of patisiran, it is encapsulated by LNPs which protect the siRNA molecules from degradation. It adsorbs apolipoprotein E (ApoE) in the blood circulation and binds to low-density lipoprotein receptors (LDL-R) on hepatocytes [30]. Upon entry into hepatocytes via receptor-mediated endocytosis, the acidic pH disrupts the lipid bilayer, releasing siRNA into the cytoplasm. Vutrisiran, on the other hand, is conjugated by GAINAc, which binds to ASGPR and triggers clathrin-mediated endocytosis.

### Mechanism of action

**Fig. 2:**
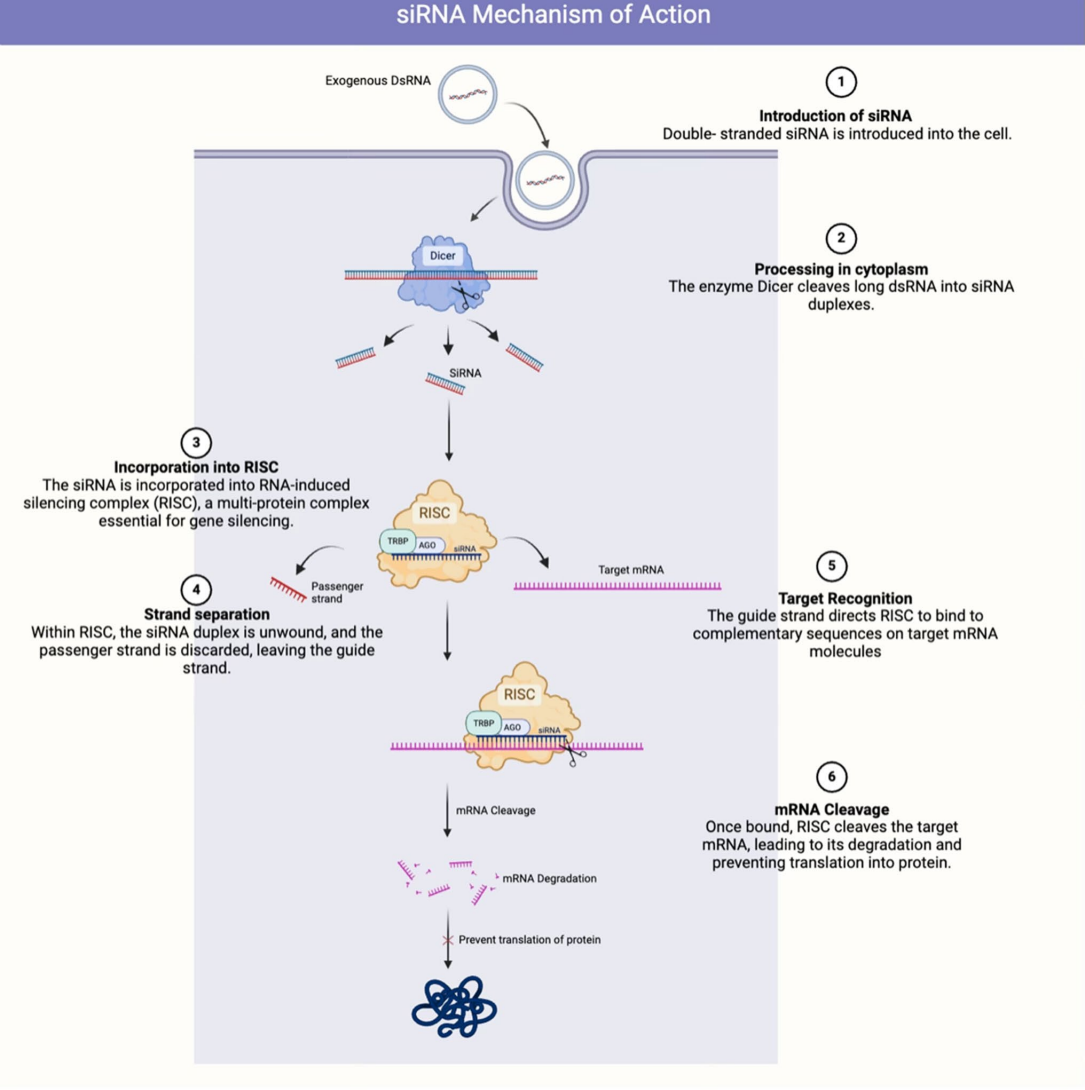
Mechanism of action of small interfering RNA (siRNA) therapy

siRNA therapy uses the cellular RNA interference (RNAi) pathway to silence gene expression (Fig. 2) [31]. siRNAs are double-stranded RNA molecules that specifically bind to the messenger RNA (mRNA) of a target gene, the TTR gene, marking it for degradation by the RNA-induced silencing complex (RISC) [32]. This prevents the translation of TTR protein and hence reduces its synthesis [33, 34].

### Revusiran

The ENDEAVOUR trial (ClinicalTrials.gov number, NCT02319005) is one of the first, phase 3, multicenter, randomized, double-blind, placebo-controlled trial that investigated the use of revusiran in ATTR-CM patients [35]. Participants were randomized in a 2: 1 ratio to receive revusiran (*n* = 140) or placebo (*n* = 66). As aforementioned, this trial was terminated prematurely, after a median Revusiran exposure of approximately 6 months due to an excessive on-treatment mortality in the treatment arm relative to the placebo arm (12.9% in the Revusiran group vs. 3.0% in the placebo group). Investigators attributed most deaths to cardiovascular causes related to heart failure. A post hoc analysis revealed that these patients were predominantly aged 75 years or older and had advanced heart failure at baseline. Furthermore, adverse events, including peripheral neuropathy, hepatic issues, and renal complications, were more frequently observed in the revusiran group.

### Patisiran

Patisiran was first studied under the APOLLO trial (ClinicalTrials.gov number, NCT01960348) in 225 patients with ATTRv-PN and a prespecified cardiac subpopulation, consisting of 126 individuals with left ventricular wall thickness ≥ 13 mm without history of aortic valve disease and hypertension). In this latter group, it was found that patisiran significantly reduced mean left ventricular wall thickness, global longitudinal strain, and NT-proBNP levels at 18 months compared to placebo. Furthermore, the composite outcome of cardiac hospitalizations and all-cause mortality was 46% lower with patisiran (HR 0.53; 95% CI 0.28–1.01). The APOLLO-B trial (ClinicalTrials.gov number, NCT03997383) is a randomized, international, multicenter, phase 3, double-blind and placebo-controlled trial investigating the use of patisiran in ATTR-CM patients [9]. Eligibility criteria included adults aged 18–85 with biopsied-confirmed or fulfilled non-invasive diagnostic criteria of ATTR cardiac amyloidosis and heart failure, while key exclusions were advanced NYHA class and severe polyneuropathy. Patisiran was administered once every 3 weeks for 12 months to the intervention group (Table 1). Primary endpoint was functional capacity measured using the six-minute walk test. Secondary endpoints included change in Kansas City Cardiomyopathy Questionnaire–Overall Summary (KCCQ-OS) score, composite outcome involving all-cause mortality, cardiovascular events and change in six-minute walking distance at 12 months, another composite outcome consisting of all-cause mortality, all-cause hospitalizations, or urgent visits for heart failure hospitalization. Change in serum TTR level from baseline to month 12 was also explored. A total of 360 patients were randomized to receive patisiran (*n* = 181) or placebo (*n* = 179). The patisiran group demonstrated a significantly smaller decline in the 6-min walk distance at 12 months compared to placebo (median difference: 14.69 m; 95% CI 0.69 to 28.69; *P* = 0.02). Additionally, the KCCQ-OS score improved in the patisiran group and decreased in the placebo group (mean difference: 3.7 points; 95% CI 0.2 to 7.2; *P* = 0.04).

**Table 1:** Overview of gene-modulating therapies in ATTR-CM

Therapy	Class of medications	Mode, dose, and frequency of administration	Premedication	Vitamin A supplementation	Trial name	Population (intervention vs. control)	Key findings	Safety profile	Monitoring requirements
Eplontersen	Antisense oligonucleotide therapy	Monthly self-administered subcutaneous autoinjector (45 mg)	No	RDA or up to 3000 IU/day	NEURO-TTRansform (NCT04136184) [25]	Subgroup of ATTRv with polyneuropathy and cardiomyopathy (49 vs. 30)	Improved left ventricular ejection fraction (+ 4.3%) and stroke volume (+ 10.64 mL) at week 65	Generally well-tolerated; minor injection site reactions noted	None beyond standard care
Patisiran	siRNA therapy	Intravenously every 3 weeks at 0.3 mg/kg (for patients < 100 kg) or 30 mg fixed dose (≥ 100 kg)	IV dexamethasone, oral acetaminophen, and IV H1/H2 blockers	Daily supplement containing the locally RDA of Vitamin A	APOLLO-B (NCT03997383) [9]	ATTR-CM (181 vs. 179)	Smaller decline in 6-min walk distance (+ 14.69 m, *P* = 0.02) and improved quality-of-life scores	Mild to moderate infusion-related reactions reported	Not required
Vutrisiran	siRNA therapy	Subcutaneously every 3 months (25 mg)	No	Daily supplement containing the locally RDA of vitamin A	HELIOS-B (NCT04153149) [6]	ATTR-CM (326 vs. 329)	28% reduction in composite endpoint of all-cause mortality and cardiovascular events (HR 0.72)	High rates of adverse events (99%) but similar to placebo; cardiac failure was the most common serious AE	Not required
Nexiguran Ziclumeran (NTLA-2001)	CRISPR-Cas9 technology	single intravenous infusion (0.7 mg/kg)	Oral dexamethasone 8 to 24 h prior, followed by IV steroids, H1 and H2 blocker 1 to 2 h prior	Daily supplement containing the locally RDA of vitamin A	Phase 1 (NCT04601051) [41]	ATTR-CM (36 intervention, no control arm)	Sustained 90% reduction in TTR levels at 12 months; maintained at 24 months in long-term follow-up	94% reported adverse events; 39% experienced serious adverse events including heart failure and arrhythmia	Vital signs, EKG, Echocardiogram, liver function (AST, ALT), vitamin A and thyroid function, ophthalmic examination, electrolytes, glucose and hematologic parameters, coagulation profile

*ALT* alanine aminotransferase, *AST* aspartate aminotransferase, *ATTR-CM* transthyretin amyloid cardiomyopathy,* EKG *Eelectrocardiogram, *H1/H2* blockers histamine-1/histamine-2 receptor antagonists, *IU* international units, *IV* intravenous, *RDA* recommended dietary allowance, *SC* subcutaneous

In the APOLLO-B open-label extension (OLE), patients who initially received placebo during the 12-month double-blind (DB) period and later switched to patisiran demonstrated slower disease progression or stabilization across key clinical and biomarker measures including 6MWT, KCCQ-OS, NT-proBNP, and troponin I [10]. Importantly, long-term patisiran use remained well-tolerated in this OLE study, with no new safety concerns observed.

### Vutrisiran

The HELIOS-B trial (ClinicalTrials.gov number, NCT04153149) is a phase 3 multicenter, double-blinded, placebo-controlled, randomized controlled trial that investigated the efficacy and safety of vutrisiran in ATTR-CM patients [6]. Patients aged 18–85 with either wtATTR-CM or ATTRv-CM with at least one HF hospitalization or clinical evidence of HF were included. Notable exclusion included NYHA class III or IV. In terms of trial design, patients were randomized to receive either vutrisiran every 12 weeks for up to 36 months or placebo (Table 1). A total of 655 patients were randomly assigned to the intervention group (vutrisiran, *n* = 326) and control group (placebo, *n* = 329). Notably, approximately 40% of patients were receiving tafamidis at baseline in each arm. Vutrisiran led to a 28% reduction in the primary endpoint, a composite of all-cause mortality and recurrent cardiovascular events (HR 0.72; 95% CI, 0.56–0.93) compared to placebo over 36 months. Furthermore, vutrisiran led to a rapid and sustained reduction in serum TTR levels (mean trough % reduction, 81.0%; 95% CI, 79.0–83.0) at 30 months. In a prespecified subgroup analysis, vutrisiran reduced the primary endpoint by 33% in the monotherapy population (without tafamidis use) and by 21% in the combination therapy population (vutrisiran + baseline tafamidis use) [6]. In terms of safety endpoint, 99% of patients receiving vutrisiran and 98% of patients receiving placebo experienced at least one adverse event. Safety profiles were comparable (serious adverse events: 62% vutrisiran vs. 67% placebo; cardiac failure: 12% vs. 17%), with low discontinuation rates (3% vs. 4% respectively).

Following the primary study, prespecified subanalysis of HELIOS-B was performed to assess the effect of vutrisiran on outpatient worsening of heart failure, defined as initiation or intensification of oral diuretics [36]. It was found that patients who experienced worsening HF had more than twofold higher risk of composite of all-cause mortality and recurrent cardiovascular events. Vutrisiran resulted in a 27% risk reduction in first outpatient worsening HF, 34% risk reduction in recurrent outpatient worsening HF, and 32% risk reduction in the expanded composite outcome of all-cause mortality, recurrent cardiovascular events, and recurrent outpatient worsening HF [36]. When stratified by baseline tafamidis use, there is a similar pattern of risk reduction across the endpoint including time to first outpatient worsening HF and recurrent outpatient worsening HF. However, the magnitude of risk reduction appeared to be greater in the monotherapy population compared to the combination therapy population (37% vs. 11% risk reduction in first outpatient worsening HF; 42% vs. 21% risk reduction in recurrent outpatient worsening HF, respectively) [36].

### Challenges of siRNA therapies

Like ASO, siRNA therapies face delivery and specificity challenges. Unmodified siRNA rapidly degrades in circulation and undergoes renal clearance due to its small size (~ 13 kDa) and susceptibility to nucleases, necessitating chemical stabilization. Patisiran contained siRNA that was chemically modified with addition of 11 sugar residues with 2′-methoxy groups and 4 residues of 2′-deoxythymidine and was encapsulated with lipid excipients to enhance stability and reduce off-target effects [30, 37]. Vutisiran employs 2′-fluoro and 2′-O-methoxyethyl modifications on its siRNA strands, along with phosphorothioate linkages, to enhance nuclease resistance, mRNA binding affinity, and pharmacokinetics [38, 39]. Its trivalent GalNAc conjugation enables targeted liver delivery via ASGPR [28].

## CRISPR-Cas9 gene editing

### Mechanism of entry

NTLA-2001 employs LNP that encapsulates Cas9 mRNA and singe-guided RNA (sgRNA) [7]. As mentioned before, LNPs adsorb ApoE which facilitates LDL-R mediated uptake to hepatocytes [40]. After endocytosis, endosomal escape releases CRISPR components into the cytoplasm [40].

### Mechanism of action

**Fig. 3:**
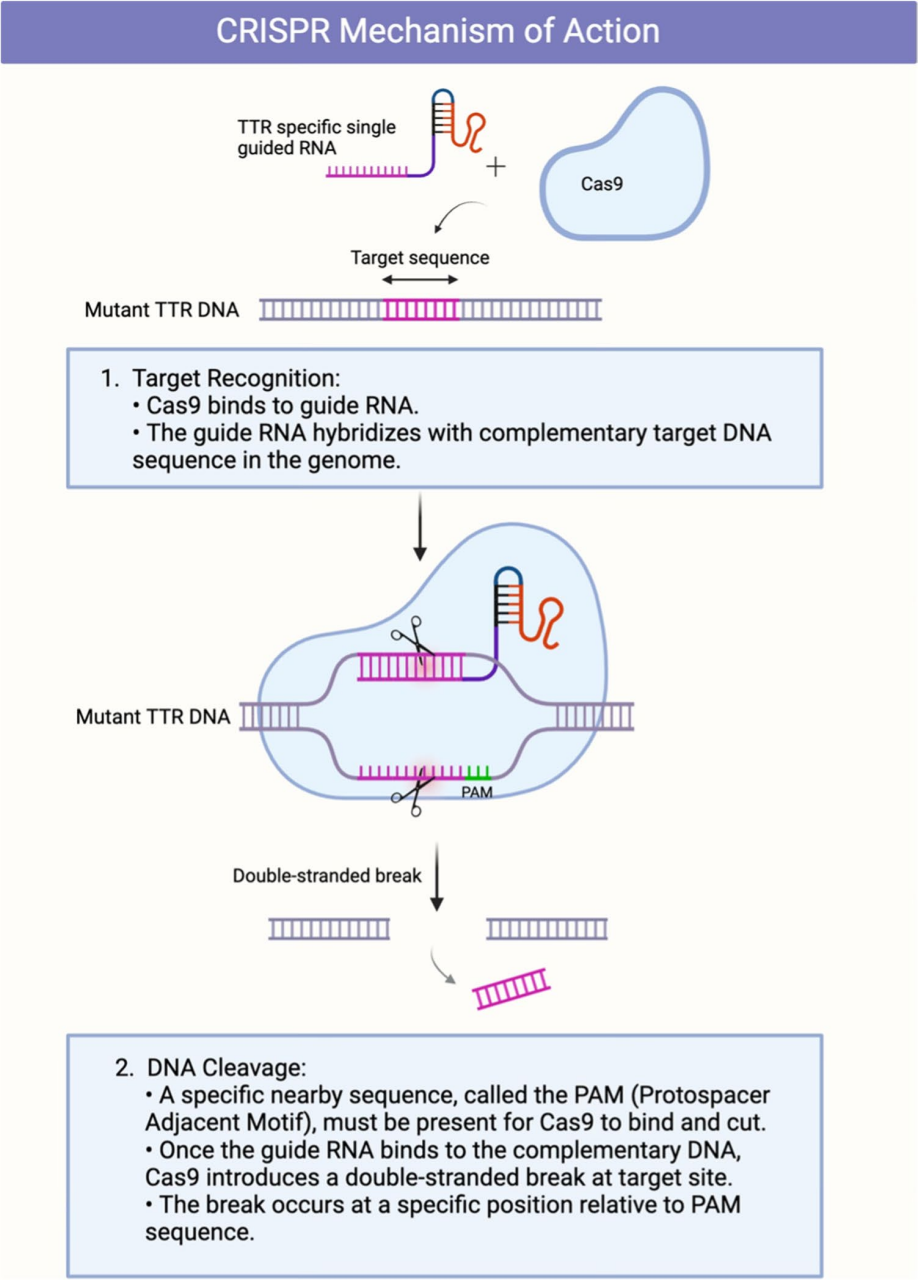
Mechanism of action of CRISPR-Cas9 gene editing

CRISPR-Cas9 technology takes a more pragmatic and permanent approach by directly editing the TTR gene itself. This system relies on two components: a sgRNA that targets the specific DNA sequence of the TTR gene and the Cas9 protein, which creates a double-strand break at the targeted site (Fig. 3) [7]. Cellular repair mechanisms then either disrupt the gene via non-homologous end joining (NHEJ) or precisely edit it through homology-directed repair (HDR), permanently disrupting TTR production.

### Nex-z

Fontana and colleagues conducted a phase 1, open-label trial (ClinicalTrials.gov number, NCT04601051) to evaluate the safety and pharmacodynamic effects of nexiguran ziclumeran (nex-z), a CRISPR-Cas9-based therapy for ATTR-CM [41]. Thirty-six patients (median age 78 years, 97% male, 78% White, and 69% with wild-type ATTR-CM) received a single intravenous infusion of nex-z and were followed for 12 months. The primary outcome was safety and TTR suppression, measured by serum TTR levels as a surrogate marker. The study demonstrated a significant reduction in serum TTR levels, with an 89% decrease at 28 days and a sustained 90% reduction at 12 months. Additionally, this reduction was maintained at 24 months in all 11 patients who completed the two-year follow-up. In terms of safety endpoints, at least one adverse event was reported in 34 out of 36 patients (94%), with 14 patients (39%) experiencing serious adverse events. Serious events included 4 hospitalizations due to heart failure, 2 cases of arrhythmia, and 1 case involving both conditions. One death occurred during the study, involving a 76-year-old male patient with a history of ischemic heart disease and V122I variant ATTR-CM, who eventually died due to ischemic heart disease. Despite these adverse events, the therapy was well-tolerated, with no permanent treatment discontinuations directly attributed to nex-z.

#### Challenges of CRISPR-Cas9 gene editing therapy

### Specificity and off-target risks

The rigorous off-target assessment in preclinical and clinical studies demonstrates exceptional specificity for the *TTR* locus [7]. Using a multi-platform approach combining Cas-OFFinder in silico prediction, GUIDE-Seq (genome-wide unbiased identification of DSBs enabled by sequencing), and SITE-Seq (selective enrichment and identification of tagmented DNA ends) in human hepatocytes, researchers identified only seven potential off-target sites; all in the noncoding regions. rhAMPseq analysis at 3 times the therapeutic EC90 concentration further showed no detectable off-target editing at these loci, confirming sgRNA’s precision even under supratherapeutic conditions [7]. This specificity stems from the optimized sgRNA design pipeline, which excluded candidates with homology to pathogenic TTR variants or single-nucletotide polymorphisms while prioritizing sequences with minimal off-target potential through in vitro genotoxicity screens [7].

### Long-term safety and ethical concerns

CRISPR-Cas9 therapies, particularly concerning irreversible genomic edits, warrant consideration over its long-term safety and ethical boundaries. Preclinical studies reveal risks of chromosomal rearrangements and large deletions at on-target sites, as seen in *NCF1* gene editing [42], though none were observed in NTLA-2001 trial at 24 months [7]. These errors, though rare, could predispose to malignancies over decades, which are areas of critical concern for younger patients with longer lifespans [43]. Conversely, older patients (median age 78 in NTLA-2001 trial) may prioritize rapid disease stabilization over hypothetical delayed risks, given their shorter life expectancy [41]. Ethical frameworks must balance this risk–benefit asymmetry. CRISPR’s irreversibility demands lifelong surveillance of hepatic dysfunction, oncogenesis, or immune responses to Cas9. Furthermore, stringent post-marketing studies remain essential to validate durability and safety beyond 5 to 10 years. These considerations underline the need for cautious and responsible integration of CRISPR-Cas9 into clinical practice [43]. CRISPR’s appeal may skew toward older cohorts, while younger patients await refinements in precision to mitigate long-term uncertainties.

## Potential of combination or switching therapies

The potential for safe yet synergistic effects through the combination of TTR stabilizers and gene-modulating therapies represents an important step forward in the management of ATTR-CM. This was evidenced by the HELIOS-B trial that included a subgroup analysis of patients receiving tafamidis at baseline in combination with the investigational drug, vutrisiran [6]. Although the study was not explicitly designed with sufficient power to evaluate the efficacy of combination therapy, the exploratory, prespecified subgroup analysis showed a reduction of primary endpoint in patients receiving combination therapy. Similar risk reduction was also shown in the subanalysis of HELIOS-B which studied the risk of first and recurrent outpatient worsening HF [36]. Switching therapies may also be warranted in patients with disease progression while on monotherapy but data within this domain remained sparse. In the NEUTRO-TTRansform trial, a small subset of patients randomized to inortersen were switched to receiving eplontersen [44]. Notably, the absolute change in serum TTR was greater after switching to eplontersen. Additionally, the reduction in platelet count with inotersen showed improvement to baseline level during eplotersen treatment [44]. However, the Institute for Clinical and Economic Review (ICER) reported that there was insufficient evidence for routine combination therapy, highlighting the need for dedicated trials comparing different sequencing strategies [45]. These early findings highlight both the therapeutic potential and current evidence gaps in optimizing multimodal approaches for ATTR-CM.

## Cost and accessibility of therapies

Cost and accessibility of these therapies present significant barriers, where the annual prices of these medications far exceeding the ICER’s cost-effectiveness thresholds [45]. The current wholesale acquisition cost of eplontersen and vutrisiran are approximately $450,000 to $500,000 per year [46, 47]. Similarly, patisiran requires 90–95% price reduction to align with value-based pricing [45]. These exorbitant costs exacerbate existing disparities, as Medicare beneficiaries face additional annual out-of-pocket expenses after accounting for cost post-inflation reduction act (IRA). Globally, access is largely restricted to high-income nations, as low/middle-income countries (LMICs) lack diagnostic infrastructure, face prices dwarfing average incomes, thus potentially perpetuating a survival difference [48]. Strategies to mitigate these barriers include policy-driven price negotiation by leveraging IRA’s provisions, global-tiered pricing (differential pricing based on countries’ income status), risk-sharing agreements tying payments to real-world pragmatic outcomes, and expanding telemedicine to bridge rural and socioeconomic gaps [49–51].

## Future outlook

To date, TTR stabilizers remain the only class of medications approved by the FDA for the treatment of ATTR-CM. Tafamidis, the first TTR stabilizer, received approval in 2019. Recently, acoramidis became the second TTR stabilizer to gain FDA approval, following the positive results of the phase 3 ATTRibute-CM study (ClinicalTrials.gov number NCT03860935) [52]. In contrast, none of the gene-modulating therapies have yet been approved for use in patients with cardiac amyloidosis. Among these, patisiran is currently approved for the treatment of polyneuropathy in ATTRv patients. However, its expansion to include ATTR-CM was denied by the FDA due to insufficient evidence of clinically meaningful benefits. Notably, it was centered on concerns about only modest functional benefits observed over 12 months (15 m improvement on the 6-minute walk test) and the lack of significant reductions in mortality or cardiovascular events [9]. Consequently, the manufacturer has ceased efforts to pursue this indication for ATTR-CM patients. Inotersen was also approved but was voluntarily discontinued by the manufacturer, while eplontersen and vutrisiran are also clinically availably for ATTR-polyneuropathy [23].

Despite these setbacks, the future of therapies for ATTR-CM remains promising. The clinical trials are yielding encouraging results, but these novel treatments require long-term surveillance for adverse effects and durability of benefit [4]. Additionally, there is a notable lack of direct comparisons among the different therapeutic modalities, representing a critical avenue for future research. To address this gap, network meta-analyses integrating data from different RCTs could indirectly compared the treatment effects of each of these therapies using validated endpoints like 6-minute walk distance and cardiac biomarkers. Concurrently, longitudinal real-world registries could further explore the sustained efficacy and unmask rare adverse events, particularly for the irreversible CRISPR-mediated TTR suppression. Biomarker-stratified trials assessing treatment sequences could further inform sequencing strategies, such as prioritizing CRISPR in patients with rapid TTR rebound after siRNA/ASO therapy. These strategies, combined with the ongoing trials evaluating eplontersen and nex-z (Table 2), highlight the dynamic pipeline of delivering precision therapies for this challenging condition.

**Table 2:** Summary of ongoing clinical trials

Study title	Therapy	ClinicalTrials.gov Identifier	Study design	Estimated sample size	Primary outcome(s)	Follow-up period
CARDIO-TTRansform: A Study to Evaluate the Efficacy and Safety of Eplontersen in Participants With Transthyretin-Mediated Amyloid Cardiomyopathy [53]	Eplontersen	NCT04136171	Phase 3, randomized, double-blind, placebo-controlled, multicenter	1400 Intervention: 716 Placebo: 716	Composite of cardiovascular (CV) mortality and recurrent CV clinical events	140 weeks with long-term extension up to 36 months
EPIC-ATTR: A Study to Evaluate the Effect of Eplontersen on the Transthyretin Reduction and Long-term Safety in Chinese Subjects With Transthyretin Amyloid Cardiomyopathy [54]	Eplontersen	NCT06194825	Phase 3, open-label, multicenter	64 Intervention = 48 Placebo = 16	Percentage change from baseline in serum TTR concentration up to week 24	Initial 24-week, double-blind and placebo-controlled treatment phase, followed by an 80-week open-label extension treatment phase
MAGNITUDE: A Phase 3 Study of NTLA-2001 in Participants With Transthyretin Amyloidosis With Cardiomyopathy [55]	NTLA-2001	NCT06128629	Phase 3, randomized, double-blind, placebo-controlled, multicenter	765 Intervention = 510 Placebo = 255	Composite of CV mortality and CV events	Up to 48 months

## Conclusion

Gene-modulating therapies represent a transformative advancement in the management of ATTR-CM, offering disease-modifying strategies that targets the root cause of the disease. While TTR stabilizers remain the cornerstone of FDA-approved treatment, emerging approaches such as RNA interference and CRISPR-Cas9 gene editing have demonstrated substantial reductions in TTR levels and promising clinical outcomes in trials. The HELIOS-B trial highlights the potential of combination therapy. However, long-term safety, durability of efficacy, and comparative studies among these therapies are critical for guiding their future use. The dynamic evolution of ATTR-CM therapies highlights the potential for more effective treatments, paving the way for improved patient outcomes in this population. Cost and accessibility of therapies remain critical challenges. Value-based pricing reforms, global-tiered pricing models, risk sharing agreements, and telemedicine expansion are potential avenue to mitigate the observed disparities.

## Data Availability

No datasets were generated or analysed during the current study.

## References

[1] Writing Committee; Kittleson MM, Ruberg FL, Ambardekar AV, Brannagan TH, Cheng RK et al (2023) 2023 ACC expert consensus decision pathway on comprehensive multidisciplinary care for the patient with cardiac amyloidosis: a report of the American College of Cardiology Solution Set Oversight Committee. J Am Coll Cardiol 81(11):1076–1126 10.1016/j.jacc.2022.11.022 36697326

[2] Griffin JM, Rosenthal JL, Grodin JL, Maurer MS, Grogan M, Cheng RK (2021) ATTR amyloidosis: current and emerging management strategies: JACC: cardiooncology state-of-the-art review. JACC CardioOncol 3(4):488–505 34729521 10.1016/j.jaccao.2021.06.006 PMC8543085

[3] Ruberg FL, Grogan M, Hanna M, Kelly JW, Maurer MS (2019) Transthyretin amyloid cardiomyopathy: JACC state-of-the-art review. J Am Coll Cardiol 73(22):2872–2891 31171094 10.1016/j.jacc.2019.04.003 PMC6724183

[4] Merlini G (2023) A step forward in solving amyloidosis. N Engl J Med 389(17):1615–1617 37888921 10.1056/NEJMe2309308

[5] Tschope C, Elsanhoury A. Treatment of transthyretin amyloid cardiomyopathy: the current options, the future, and the challenges. J Clin Med. 2022;11(8). 10.3390/jcm11082148 PMC9031576 35456241

[6] Fontana M, Berk JL, Gillmore JD, Witteles RM, Grogan M, Drachman B, et al. Vutrisiran in patients with transthyretin amyloidosis with cardiomyopathy. N Engl J Med. 2024. 10.1056/NEJMc2501792 40334172

[7] Gillmore JD, Gane E, Taubel J, Kao J, Fontana M, Maitland ML et al (2021) CRISPR-Cas9 in vivo gene editing for transthyretin amyloidosis. N Engl J Med 385(6):493–502 34215024 10.1056/NEJMoa2107454

[8] Almeida M, Ranisch R (2022) Beyond safety: mapping the ethical debate on heritable genome editing interventions. Humanities and Social Sciences Communications 9(1):139

[9] Maurer MS, Kale P, Fontana M, Berk JL, Grogan M, Gustafsson F et al (2023) Patisiran treatment in patients with transthyretin cardiac amyloidosis. N Engl J Med 389(17):1553–1565 37888916 10.1056/NEJMoa2300757 PMC10757426

[10] Hung RR, Correia Ed, Berk JL, Drachman B, Fontana M, Gillmore JD, et al. Abstract 13273: APOLLO-B, a study of patisiran in patients with transthyretin cardiac amyloidosis: primary long-term results from the open-label extension period. Circulation. 2023;148(Suppl_1):A13273-A.

[11] Buxbaum JN, Reixach N (2009) Transthyretin: the servant of many masters. Cell Mol Life Sci 66(19):3095–3101 19644733 10.1007/s00018-009-0109-0 PMC4820353

[12] Brito D, Albrecht FC, de Arenaza DP, Bart N, Better N, Carvajal-Juarez I et al (2023) World Heart Federation consensus on transthyretin amyloidosis cardiomyopathy (ATTR-CM). Glob Heart 18(1):59 37901600 10.5334/gh.1262 PMC10607607

[13] Hund E, Linke RP, Willig F, Grau A (2001) Transthyretin-associated neuropathic amyloidosis. Pathogenesis and treatment Neurology 56(4):431–435 11261421 10.1212/wnl.56.4.431

[14] Maurer MS, Hanna M, Grogan M, Dispenzieri A, Witteles R, Drachman B et al (2016) Genotype and phenotype of transthyretin cardiac amyloidosis: THAOS (Transthyretin Amyloid Outcome Survey). J Am Coll Cardiol 68(2):161–172 27386769 10.1016/j.jacc.2016.03.596 PMC4940135

[15] Jacobson DR, Pastore RD, Yaghoubian R, Kane I, Gallo G, Buck FS et al (1997) Variant-sequence transthyretin (isoleucine 122) in late-onset cardiac amyloidosis in Black Americans. N Engl J Med 336(7):466–473 9017939 10.1056/NEJM199702133360703

[16] Iorio A, De Lillo A, De Angelis F, Di Girolamo M, Luigetti M, Sabatelli M et al (2017) Non-coding variants contribute to the clinical heterogeneity of TTR amyloidosis. Eur J Hum Genet 25(9):1055–1060 28635949 10.1038/ejhg.2017.95 PMC5558178

[17] Griffin JM, Rosenblum H, Maurer MS (2021) Pathophysiology and therapeutic approaches to cardiac amyloidosis. Circ Res 128(10):1554–1575 33983835 10.1161/CIRCRESAHA.121.318187 PMC8561842

[18] Brannagan TH 3rd, Berk JL, Gillmore JD, Maurer MS, Waddington-Cruz M, Fontana M et al (2022) Liver-directed drugs for transthyretin-mediated amyloidosis. J Peripher Nerv Syst 27(4):228–237 36345805 10.1111/jns.12519 PMC10100204

[19] Viney NJ, Guo S, Tai LJ, Baker BF, Aghajan M, Jung SW et al (2021) Ligand conjugated antisense oligonucleotide for the treatment of transthyretin amyloidosis: preclinical and phase 1 data. ESC Heart Fail 8(1):652–661 33283485 10.1002/ehf2.13154 PMC7835591

[20] Benson MD, Waddington-Cruz M, Berk JL, Polydefkis M, Dyck PJ, Wang AK et al (2018) Inotersen treatment for patients with hereditary transthyretin amyloidosis. N Engl J Med 379(1):22–31 29972757 10.1056/NEJMoa1716793 PMC12611561

[21] Gales L. Tegsedi (inotersen): an antisense oligonucleotide approved for the treatment of adult patients with hereditary transthyretin amyloidosis. Pharmaceuticals (Basel). 2019;12(2). 10.3390/ph12020078 PMC6631675 31117178

[22] 24 month open label study of the tolerability and efficacy of inotersen in TTR amyloid cardiomyopathy patients: ClinicalTrials.gov; 2018 [updated 2018/10/12]. Available from: https://clinicaltrials.gov/ct2/show/NCT03702829.

[23] Akcea T. TEGSEDI REMS (risk evaluation and mitigation strategy) program - Tegsedi REMS 2025 [Available from: https://www.tegsedirems.com.

[24] Coelho T, Marques W Jr, Dasgupta NR, Chao CC, Parman Y, Franca MC Jr et al (2023) Eplontersen for hereditary transthyretin amyloidosis with polyneuropathy. JAMA 330(15):1448–1458 37768671 10.1001/jama.2023.18688 PMC10540057

[25] Masri A, Maurer MS, Claggett BL, Kulac I, Waddington Cruz M, Conceicao I et al (2024) Effect of eplontersen on cardiac structure and function in patients with hereditary transthyretin amyloidosis. J Card Fail 30(8):973–980 38065307 10.1016/j.cardfail.2023.11.016

[26] Miller CM, Tanowitz M, Donner AJ, Prakash TP, Swayze EE, Harris EN et al (2018) Receptor-mediated uptake of phosphorothioate antisense oligonucleotides in different cell types of the liver. Nucleic Acid Ther 28(3):119–127 29425080 10.1089/nat.2017.0709 PMC6037193

[27] Baker BF, Xia S, Partridge W, Kwoh TJ, Tsimikas S, Bhanot S et al (2023) Integrated assessment of phase 2 data on GalNAc(3)-conjugated 2’-O-methoxyethyl-modified antisense oligonucleotides. Nucleic Acid Ther 33(1):72–80 36454263 10.1089/nat.2022.0044 PMC10623620

[28] Debacker AJ, Voutila J, Catley M, Blakey D, Habib N (2020) Delivery of oligonucleotides to the liver with GalNAc: from research to registered therapeutic drug. Mol Ther 28(8):1759–1771 32592692 10.1016/j.ymthe.2020.06.015 PMC7403466

[29] Wada F, Yamamoto T, Ueda T, Sawamura M, Wada S, Harada-Shiba M et al (2018) Cholesterol-GalNAc dual conjugation strategy for reducing renal distribution of antisense oligonucleotides. Nucleic Acid Ther 28(1):50–57 29360004 10.1089/nat.2017.0698

[30] Urits I, Swanson D, Swett MC, Patel A, Berardino K, Amgalan A et al (2020) A review of patisiran (ONPATTRO®) for the treatment of polyneuropathy in people with hereditary transthyretin amyloidosis. Neurol Ther 9(2):301–315 32785879 10.1007/s40120-020-00208-1 PMC7606409

[31] Dana H, Chalbatani GM, Mahmoodzadeh H, Karimloo R, Rezaiean O, Moradzadeh A et al (2017) Molecular mechanisms and biological functions of siRNA. Int J Biomed Sci 13(2):48–57 28824341 PMC5542916

[32] Chery J (2016) RNA therapeutics: RNAi and antisense mechanisms and clinical applications. Postdoc J 4(7):35–50 27570789 10.14304/surya.jpr.v4n7.5 PMC4995773

[33] Leung AK, Tam YY, Cullis PR (2014) Lipid nanoparticles for short interfering RNA delivery. Adv Genet 88:71–110 25409604 10.1016/B978-0-12-800148-6.00004-3 PMC7149983

[34] Springer AD, Dowdy SF (2018) GalNAc-siRNA conjugates: leading the way for delivery of RNAi therapeutics. Nucleic Acid Ther 28(3):109–118 29792572 10.1089/nat.2018.0736 PMC5994659

[35] Judge DP, Kristen AV, Grogan M, Maurer MS, Falk RH, Hanna M et al (2020) Phase 3 multicenter study of revusiran in patients with hereditary transthyretin-mediated (hATTR) amyloidosis with cardiomyopathy (ENDEAVOUR). Cardiovasc Drugs Ther 34(3):357–370 32062791 10.1007/s10557-019-06919-4 PMC7242280

[36] Fontana M, Maurer Mathew S, Gillmore Julian D, Bender S, Aldinc E, Eraly Satish A, et al. Outpatient worsening heart failure in patients with transthyretin amyloidosis with cardiomyopathy in the HELIOS-B trial. Journal of the American College of Cardiology.0(0). 10.1016/j.jacc.2024.11.015 39566871

[37] Dixon S, Kang X, Quan D (2023) Practical guidance for the use of patisiran in the management of polyneuropathy in hereditary transthyretin-mediated amyloidosis. Ther Clin Risk Manag 19:973–981 38047038 10.2147/TCRM.S361706 PMC10691373

[38] Gangopadhyay S, Gore KR (2022) Advances in siRNA therapeutics and synergistic effect on siRNA activity using emerging dual ribose modifications. RNA Biol 19(1):452–467 35352626 10.1080/15476286.2022.2052641 PMC8973385

[39] Setten RL, Rossi JJ, Han S-p. The current state and future directions of RNAi-based therapeutics. Nature Reviews Drug Discovery. 2019;18(6):421–46. 10.1038/s41573-019-0017-4 30846871

[40] Zhang T, Yin H, Li Y, Yang H, Ge K, Zhang J, et al. Optimized lipid nanoparticles (LNPs) for organ-selective nucleic acids delivery in vivo. iScience. 2024;27(6):109804. 10.1016/j.isci.2024.109804 PMC11103379 38770138

[41] Fontana M, Solomon SD, Kachadourian J, Walsh L, Rocha R, Lebwohl D, et al. CRISPR-Cas9 gene editing with nexiguran ziclumeran for ATTR cardiomyopathy. N Engl J Med. 2024. 10.1056/NEJMoa2412309 39555828

[42] Raimondi F, Siow KM, Wrona D, Fuster-García C, Pastukhov O, Schmitz M et al (2024) Gene editing of NCF1 loci is associated with homologous recombination and chromosomal rearrangements. Communications Biology 7(1):1291 39384978 10.1038/s42003-024-06959-z PMC11464842

[43] Sheridan C (2021) CRISPR therapies march into clinic, but genotoxicity concerns linger. Nat Biotechnol 39(8):897–899 34267391 10.1038/d41587-021-00017-3

[44] Conceição I, Berk JL, Weiler M, Kowacs PA, Dasgupta NR, Khella S et al (2024) Switching from inotersen to eplontersen in patients with hereditary transthyretin-mediated amyloidosis with polyneuropathy: analysis from NEURO-TTRansform. J Neurol 271(10):6655–6666 39138650 10.1007/s00415-024-12616-6 PMC11447117

[45] Wasfy JH, Winn AN, Touchette DR, Nikitin D, Shah KK, Richardson M, et al. Disease modifying therapies for the treatment of transthyretin amyloid cardiomyopathy; Draft Evidence Report. Institute for Clinical and Economic Review; 2024 2024/07/17.

[46] AMVUTTRA (vutrisiran) product characteristics. Alnylam Assist.

[47] Drug approvals monthly update: January 2024. Prime Therapeutics. 2024.

[48] Yadav H, Shah D, Sayed S, Horton S, Schroeder LF (2021) Availability of essential diagnostics in ten low-income and middle-income countries: results from national health facility surveys. Lancet Glob Health 9(11):e1553–e1560 34626546 10.1016/S2214-109X(21)00442-3 PMC8526361

[49] Kim AE, Choi DH, Chang J, Kim SH (2020) Performance-based risk-sharing arrangements (PBRSA): is it a solution to increase bang for the buck for pharmaceutical reimbursement strategy for our nation and around the world? Clin Drug Investig 40(12):1107–1113 33037566 10.1007/s40261-020-00972-w PMC7546145

[50] Butzner M, Cuffee Y (2021) Telehealth interventions and outcomes across rural communities in the United States: narrative review. J Med Internet Res 23(8):e29575 34435965 10.2196/29575 PMC8430850

[51] Garrison LP Jr, Carlson JJ, Bajaj PS, Towse A, Neumann PJ, Sullivan SD et al (2015) Private sector risk-sharing agreements in the United States: trends, barriers, and prospects. Am J Manag Care 21(9):632–640 26618366

[52] Gillmore JD, Judge DP, Cappelli F, Fontana M, Garcia-Pavia P, Gibbs S et al (2024) Efficacy and safety of acoramidis in transthyretin amyloid cardiomyopathy. N Engl J Med 390(2):132–142 38197816 10.1056/NEJMoa2305434

[53] A phase 3 global, double-blind, randomized, placebo-controlled study to evaluate the efficacy and safety of ION-682884 in patients with transthyretin-mediated amyloid cardiomyopathy (ATTR CM) [Internet]. 2019. Available from: https://clinicaltrials.gov/study/NCT04136171.

[54] A phase 3, randomized study, with initial 24-week, double-blind and placebo-controlled treatment phase, followed by an 80-week open-label extension treatment phase to evaluate the effect of eplontersen on the transthyretin reduction and long-term safety in Chinese participants with transthyretin amyloid cardiomyopathy (EPIC-ATTR) [Internet]. 2023. Available from: https://clinicaltrials.gov/study/NCT06194825.

[55] MAGNITUDE: a phase 3, multinational, multicenter, randomized, double-blind, placebo-controlled study to evaluate the efficacy and safety of NTLA-2001 in participants with transthyretin amyloidosis with cardiomyopathy (ATTR-CM) [Internet]. 2023. Available from: https://clinicaltrials.gov/study/NCT06128629.

